# Pulse Granulomas of the Gastrointestinal Tract and Gallbladder: Report of Five Cases

**DOI:** 10.1155/2017/2497945

**Published:** 2017-07-13

**Authors:** Tom C. DeRoche, Gregory A. Gates, Aaron R. Huber

**Affiliations:** ^1^Department of Pathology, Kaiser Airport Way Regional Laboratory, 13705 NE Airport Way, Suite C, Portland, OR 97230, USA; ^2^Department of Pathology, Naval Medical Center San Diego, San Diego, CA 92134, USA; ^3^University of Rochester Medical Center, 601 Elmwood Drive, P.O. Box 626, Rochester, NY 14642, USA

## Abstract

Hyaline rings with admixed multinucleated giant cells characterize pulse granulomas; the term* pulse* refers to edible seeds of legume vegetables. The etiology has been controversial, with theories including vascular degenerative changes or a reaction to vegetable material; ultrastructural studies and experimentally induced lesions in animals favor the latter. This lesion is typically seen in the oral cavity, with only rare reports in the gastrointestinal tract and gallbladder. We herein describe five cases of pulse granulomas identified in these sites. All cases contained foreign-body giant cells and vegetable debris within or near hyaline rings. Pulse granulomas may form mass lesions but are usually an incidental finding on microscopic examination. In incidentally detected cases, recognition of pulse granulomas can suggest a mural abscess, fistula, or perforation of the gut, findings which may not be grossly apparent. The presence of vegetable material in all five cases further supports an exogenous pathogenesis.

## 1. Introduction

**Figure 1:**
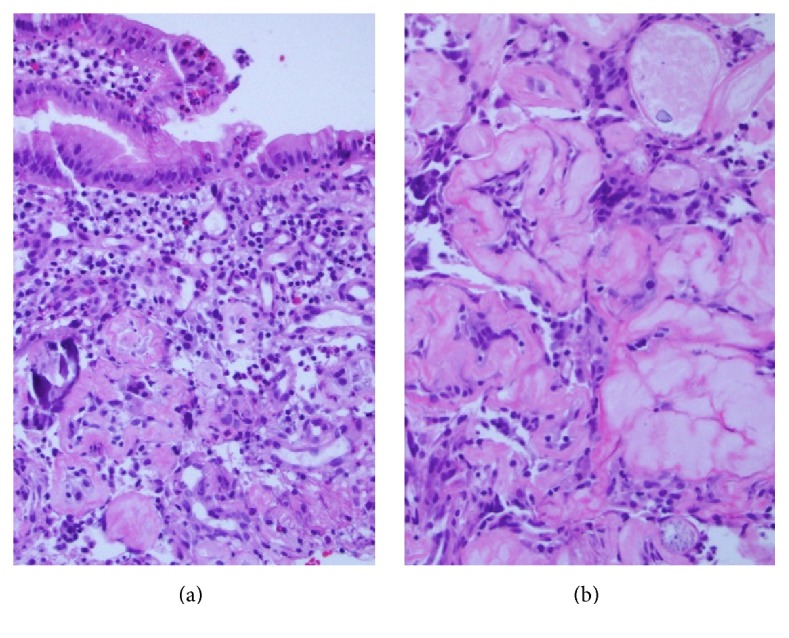
((a) and (b)) Two examples of pulse granulomas involving the gastrointestinal tract composed of the characteristic dense and eosinophilic hyaline rings ((a) H&E, original magnification ×100, and (b) H&E, original magnification ×400).

Hyaline rings with admixed multinucleated giant cells characterize pulse granulomas; the term* pulse* refers to edible seeds of legume vegetables. The etiology has been controversial, with theories including vascular degenerative changes or a reaction to vegetable material; ultrastructural studies and experimentally induced lesions in animals favor the latter. This lesion is typically seen in the oral cavity, with only rare reports in the gastrointestinal tract and gallbladder. We herein describe five cases of pulse granulomas identified in these sites. Case  1 was a 72-year-old female with a friable colonic mass suspicious for malignancy. Cases  2 and 3 were a 62-year-old female and 59-year-old male with peridiverticular pulse granulomas in the sigmoid colon. Case  4 was a 52-year-old male with pulse granulomas in appendiceal serosa and a coloenteric fistula tract secondary to perforated sigmoid diverticulitis. Case  5 was a 66-year-old male with pulse granulomas of the gallbladder wall in the setting of cholecystocolonic fistula secondary to chronic calculous cholecystitis. All of the cases exhibited convoluted hyaline rings varying in appearance from loose rings infiltrated by neutrophils to densely hyalinized, thick-walled rings (Figures 1(a) and 1(b)). All cases contained foreign-body giant cells and vegetable debris within or near hyaline rings. Pulse granulomas may form mass lesions but are usually an incidental finding on microscopic examination. In incidentally detected cases, recognition of pulse granulomas can suggest a mural abscess, fistula, or perforation of the gut, findings which may not be grossly apparent. The presence of vegetable material in all five cases further supports an exogenous pathogenesis. The cases are summarized in Table 1.

## 2. Case Series

### 2.1. Case  1

At colonoscopy, a 72-year-old female with chronic anemia of uncertain etiology was found to have a friable mass at 55 cm proximal to the anal verge. The biopsies showed hyaline rings surrounding histiocytes or multinucleated giant cells and associated with vegetable debris; the latter was seen as palely eosinophilic material with dense cell walls or refractile translucent material arranged in spirals. The hyaline rings were found in detached biopsy fragments or embedded within colonic lamina propria and granulation tissue. The adjoining colonic mucosa showed no features of chronic colitis.

### 2.2. Case  2

A 62-year-old female with a 15-year history of diverticulosis presented with a 4-day history of abdominal pain. CT scan revealed sigmoid diverticulitis with an associated abscess. The sigmoid colectomy specimen showed gross diverticular disease with a mural abscess. Histologic sections revealed diverticulosis and a diverticular abscess with organizing fibrinous serositis. Hyaline rings, multinucleated giant cells, and spherical calcifications were seen within peridiverticular muscularis propria and subserosa, associated with a reactive myofibroblastic proliferation and an infiltrate composed mainly of lymphocytes and eosinophils. Rare fragments of refractile vegetable material were identified.

### 2.3. Case  3

A 59-year-old male with a 4-year history of recurrent diverticulitis was treated with Ciprofloxacin/Flagyl as an outpatient. CT scan revealed sigmoid diverticulitis. The segmental sigmoid resection specimen showed gross diverticular disease. Histologic sections demonstrated an aggregate of hyaline rings within peridiverticular muscularis propria. These rings were loose in appearance, and many were infiltrated by neutrophils and had a wall structure recognizable as vegetable in origin. The hyaline rings were associated with dense chronic inflammation and abundant multinucleated giant cells.

### 2.4. Case  4

A 52-year-old male presented with sudden onset generalized abdominal pain, four months after laparoscopic washout and drainage of perforated sigmoid diverticulitis. Abdominal plain radiograph demonstrated pneumoperitoneum, prompting referral for sigmoid colectomy. The specimen consisted of a 9.5 cm length of sigmoid colon with an adherent 6.0 cm length of terminal ileum and a separate 5.2 × 2.5 × 1.2 cm appendix. While there was gross evidence of sigmoid diverticular disease, no fistula tract between the intestinal segments was identified. The appendix showed brown flecks involving the serosal surface but was otherwise unremarkable. On microscopic examination, the sigmoid and terminal ileum contained a coloenteric fistula tract with loosely formed hyaline rings, abundant vegetable material, and a predominantly suppurative inflammatory reaction with multinucleated giant cells. The sigmoid colon exhibited numerous diverticula, some of which contained impacted vegetable material. Sections of the appendix showed a 0.3 cm subserosal nodule composed of hyaline rings, vegetable material, and abundant inflammatory cells, similar to that seen in the coloenteric fistula tract.

### 2.5. Case  5

**Figure 2:**
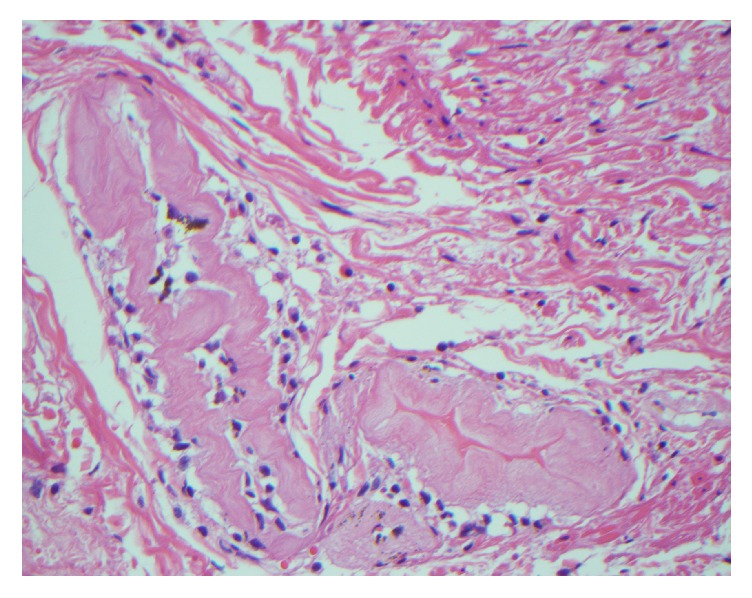
Pulse granuloma with characteristic dense and eosinophilic hyaline rings within the wall of the gallbladder (H&E, original magnification ×400).

A 66-year-old male was incidentally found to have a 0.9 cm mass in the gallbladder wall. Serial ultrasound and magnetic resonance imaging studies showed no significant interval growth over two years; some of these studies raised the possibility of a gallstone embedded in the gallbladder wall. During cholecystectomy, an apparent cholecystocolonic fistula involving the transverse colon was identified. The resected specimen consisted of an opened 4.5 × 3.0 × 3.0 cm gallbladder with slight mural thickening, a 1.5 × 1.5 × 0.5 cm fragment of fibromuscular tissue, a 1.5 cm mixed cholesterol gallstone, and a 2.5 cm length of colon with a roughened serosal surface. No fistula tract was identified grossly. Sections of the gallbladder demonstrated chronic cholecystitis. The fragment of fibromuscular tissue represented gallbladder with densely adherent colonic wall; within the muscularis propria of the gallbladder, there were hyaline rings with admixed multinucleated giant cells (Figure 2). A small, round fragment of degenerated vegetable material was identified.

## 3. Discussion

Pulse granulomas are characterized by convoluted hyaline rings with an associated foreign-body reaction, often with identifiable vegetable material; the term* pulse *refers to the edible seeds of leguminous vegetables. These lesions are most common in the oral cavity, with at least 173 reported cases; they are typically seen in the mandible of edentulous patients with dentures, in the walls of odontogenic cysts, decayed teeth or open sockets, and teeth with prior endodontic treatment [1]. The next most frequent site of involvement is the lungs, as a consequence of vegetable aspiration, and is more accurately termed "lentil pneumonia" since the morphology is somewhat different than the classic pulse granuloma. Within the lung, the morphology is usually that of a foreign-body-type giant cell reaction with or without suppurative granulomatous inflammation. Additionally, there is often an associated organizing pneumonia pattern of injury. The vegetable material is often present as small particles within the alveolar spaces. There may be concentric fibrosis around the aspirated material. The degenerated and collapsed vegetable material may appear more eosinophilic and hyalinized and resemble a pulse granuloma [2–5].

Pulse granulomas of the gastrointestinal tract and gallbladder are relatively uncommon, with only fifty and three reported cases at these respective sites. In a 1954 series of granulomas in resected peptic ulcers, Sherman and Moran illustrated one case with eosinophilic rings described as vegetable starch bodies [6]. Pereira and colleagues reported the first colonic pulse granuloma, describing a distinct 2 cm submucosal rectal mass causing clinical concern for malignancy [7]. There were three subsequent cases of colonic pulse granuloma, all found incidentally in resected sigmoid diverticular disease [8, 9]. Simsek and colleagues reported a pulse granuloma of the appendiceal serosa in the context of perforated appendicitis [10]. There have been three reports of gallbladder pulse granulomas, one in association with a cholecystoduodenal fistula related to cholecystitis and cholelithiasis [11] and one with a cholecystogastric fistula with chronic cholecystitis [12]. The most recent case was in a series of twenty-two cases, only one of which involved the gallbladder [13].

**Figure 3:**
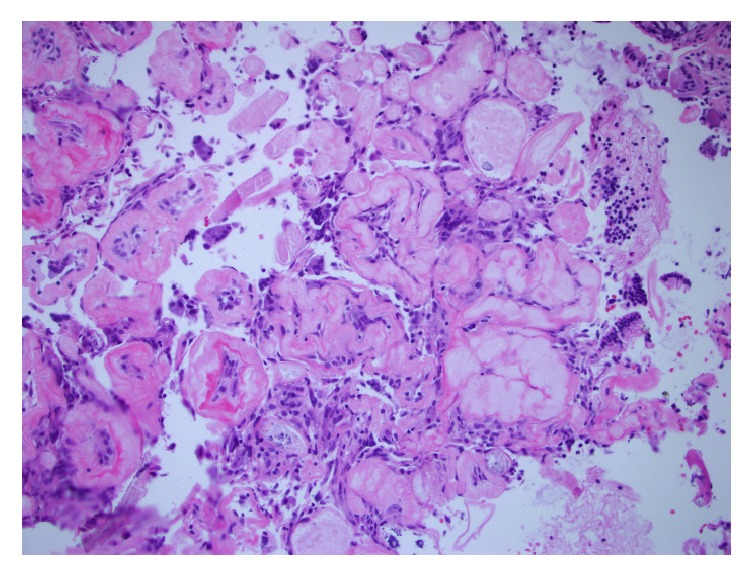
Pulse granuloma with the characteristic eosinophilic hyaline rings and spiral body (bottom left corner) which is a plant vascular structure (H&E, original magnification ×400).

The etiology of pulse granulomas has been somewhat controversial, with theories including hyaline degenerative changes in blood vessels (accounting for the prior terminology of “giant cell hyaline angiopathy”) or a reaction to exogenous vegetable material, particularly cellulose [1]. The majority of the evidence supports the latter theory. Oral pulse granulomas show similar ultrastructural features to vegetable cell walls and consist of microfibrils corresponding to cellulose, sometimes surrounded by a peripheral layer of collagen [14]. Lesions resembling pulse granulomas have been replicated in animal models by introduction of legume material into the oral cavity and skin [15, 16]; over time, the starch component is degraded while the nondigestible cellulose moiety persists [15]. Recently, a study histologically processed over 40 food items and compared the foods to patient samples and found that the material in patient samples was histologically similar to lentils and various beans [13]. Spiral bodies, which are plant vascular structures, have been identified in pulse granulomas and in one case in the current series. These spiral bodies additionally support the theory that pulse granulomas are derived from a host response to foreign plant material (Figure 3) [17]. In the current series, vegetable material was identified in all five cases, either within or adjacent to hyaline rings. The localization of these rings in peridiverticular tissue (Cases  2-3), as well as the presence of vegetable material in a colonic diverticulum, coloenteric fistula tract, and appendiceal serosa from the same case (Case  4), further suggests that pulse granulomas are related to egress of gastrointestinal luminal contents. A competing theory on the origin of pulse granulomas is degenerative changes within a vascular structure [18]; however, the above evidence argues against this theory.

While usually an incidental finding, pulse granulomas of the gastrointestinal tract and gallbladder are useful to suggest or confirm a defect in the tubular gut. In these five cases, identification of pulse granulomas confirmed the presence of mural abscesses, fistulous tracts, or frank perforation of the intestinal wall; in some of the cases, these findings were not apparent on gross pathologic examination. Additionally, Gonzalez [19] recently noted that pulse granulomas may be present in up to 10% of resections of injured small and large bowel. Pulse granulomas are also significant in that they may form mass lesions causing concern for malignancy [13]. The rectal pulse granuloma reported by Pereira and colleagues was a discrete submucosal mass clinically concerning for carcinoid tumor or lymphoid hyperplasia [7]. In our case 1, the endoscopic impression was colonic carcinoma given the presence of a friable mass in the setting of chronic anemia. Recently a sclerosing mesenteritis-like variant of mass-forming pulse granuloma has been recognized [13].

Given the distinctive appearance of the hyaline rings, the differential diagnostic considerations for pulse granuloma are fairly limited. The presumably early lesions have abundant acute inflammation, the rings have a compartmentalized structure suggestive of vegetable origin, and there is abundant free vegetable material. Cases with densely hyalinized, paucicellular rings with scant vegetable material may potentially resemble vascular amyloid deposits. In most cases, the corrugated shape and clustered distribution of the rings, presence of foreign-body giant cells, and associated vegetable material suggest the correct diagnosis. In problematic cases, special stains for amyloid would allow these entities to be distinguished. Liesegang rings may also enter the differential diagnosis, as these consist of eosinophilic material, overlap in size with pulse granulomas, and are often seen in a background of inflammatory cells and multinucleated giant cells. However, Liesegang rings are characterized by a laminated appearance with radially oriented striations. Perhaps most importantly, the granulomatous inflammatory response inherent to pulse granulomas may cause concern for Crohn's disease; this is particularly true in the gastrointestinal tract, where pulse granulomas are also associated with fistulizing disease. In peridiverticular pulse granulomas, distinction from Crohn's disease may be further confounded by architectural distortion, crypt injury with cryptolytic granulomas, and transmural inflammation related to diverticular colitis. In such cases, the recognition of diverticular disease in the segment and absence of well-formed epithelioid granulomas would prevent misclassification as Crohn's disease.

In summary, herein we have described five cases of pulse granulomas involving the gastrointestinal tract and the gallbladder. The importance of recognizing this entity, particularly in the gastrointestinal tract, is that these lesions commonly mimic malignancy and other mass-forming lesions such as sclerosing mesenteritis [13]. It is important for pathologists to be aware that pulse granulomas may affect the gastrointestinal tract and may masquerade as a malignancy and to avoid misdiagnosis of these lesions, particularly at the time of frozen section.

## Figures and Tables

**Table 1:** Case summary.

Age, gender	Location of pulse granuloma	Underlying pathology	Preoperative impression
72, F	Colon at 55 cm, lamina propria	Unknown; no resection performed	Malignancy
62, F	Sigmoid colon, muscularis propria	Diverticular disease	Diverticular abscess
52, M	Appendix and ileosigmoid fistula, subserosa	Diverticular disease	Perforated sigmoid diverticulitis
59, M	Sigmoid colon, muscularis propria	Diverticular disease	Diverticulitis
66, M	Gallbladder, muscularis propria	Cholelithiasis Chronic cholecystitis Cholecystocolonic fistula	Gallbladder mass

## References

[1] Philipsen H. P., Reichart P. A. (2010). Pulse or hyaline ring granuloma. Review of the literature on etiopathogenesis of oral and extraoral lesions. *Clinical Oral Investigations*.

[2] Knoblich R. (1969). Pulmonary granulomatosis caused by vegetable particles. So-called lentil pulse pneumonia. *American Review of Respiratory Disease*.

[3] Mukhopadhyay S., Katzenstein A.-L. A. (2007). Pulmonary disease due to aspiration of food and other particulate matter: a clinicopathologic study of 59 cases diagnosed on biopsy or resection specimens. *American Journal of Surgical Pathology*.

[4] Crome L., Valentine J. C. (1962). Pulmonary nodular granulomatosis caused by inhaled vegetable particles. *Journal of clinical pathology*.

[5] Head M. A. (1956). Foreign body reaction to inhalation of lentil soup: giant cell pneumonia. *Journal of Clinical Pathology*.

[6] Sherman F. E., Moran T. J. (1954). Granulomas of Stomach: I. Response to Injury of Muscle and Fibrous Tissue of Wall of Human Stomach. *American Journal of Clinical Pathology*.

[7] Pereira T. C., Prichard J. W., Khalid M., Medich D. S., Silverman J. F. (2001). Rectal pulse granuloma. *Archives of Pathology & Laboratory Medicine*.

[8] Zhai J., Maluf H. M. (2004). Peridiverticular colonic hyaline rings (pulse granulomas): report of two cases associated with perforated diverticula. *Annals of Diagnostic Pathology*.

[9] Stewart C. J. R., Hillery S. (2005). Peridiverticular colonic hyaline rings (pulse granulomas). *Annals of Diagnostic Pathology*.

[10] Simsek G. G., Bulus H., Guresci S. (2012). Pulse granuloma, unusual localization: appendix. *The Turkish Journal of Gastroenterology*.

[11] Lack E. E. Pathology of the pancreas, gallbladder, extrahepatic biliary tract, and ampullary region. Oxford, New York.

[12] Rhee D. D., Wu M. L. (2006). Pulse granulomas detected in gallbladder, fallopian tube, and skin. *Archives of Pathology & Laboratory Medicine*.

[13] Nowacki N. B., Arnold M. A., Frankel W. L. (2015). Gastrointestinal tract-derived pulse granulomata: clues to an underrecognized pseudotumor. *American Journal of Surgical Pathology*.

[14] Harrison J. D., Martin I. C. (1986). Oral vegetable granuloma: ultrastructural and histological study. *Journal of Oral Pathology*.

[15] Talacko A. A., Radden B. G. (1988). The pathogenesis of oral pulse granuloma: an animal model. *Journal of Oral Pathology & Medicine*.

[16] Watson R. E., Stewart C. (1991). Experimental oral foreign body reactions: vegetable materials. *Oral Surgery, Oral Medicine, Oral Pathology*.

[17] Williams A. S., Bullock M. (2015). Spiral bodies in pulse granulomas are remnant plant vascular structures. *Pathology*.

[18] Pilatti A., Patriarca C. (2013). Perianal pulse granuloma. *International Journal of Surgical Pathology*.

[19] Gonzalez R. S. (2016). Incidence of pulse granuloma in the small and large intestines. *American Journal of Surgical Pathology*.

